# Incidence of complications and secondary procedure following distal radius fractures treated by volar locking plate (VLP)

**DOI:** 10.1186/s13018-019-1344-1

**Published:** 2019-09-04

**Authors:** Yansen Li, Yanqing Zhou, Xiong Zhang, Dehu Tian, Bing Zhang

**Affiliations:** 1 Department of Foot and Ankle Surgery, The Third Hospital of Hebei Medical University, Shijiazhuang, 050051 Hebei People’s Republic of China; 2 Key Laboratory of Biomechanics of Hebei Province, Shijiazhuang, 050051 Hebei People’s Republic of China; 3 Department of Hand Surgery, The Third Hospital of Hebei Medical University, NO.139 Ziqiang Road, Shijiazhuang, 050051 Hebei People’s Republic of China

**Keywords:** Complication, Distal radius fracture, Volar locking plate, Incidence rate, Risk factors

## Abstract

**Objective:**

To investigate the incidence of postoperative overall complications or secondary procedures following distal radius fractures treated by volar locking plate (VLP)

**Methods:**

Electronic medical records (EMR) of 1152 patients with 1175 distal radius fractures treated by volar locking plate between January 2013 and September 2018 were retrospectively reviewed and the data were extracted. The picture archiving and communication system (PACS) was inquired to assess the fracture severity and to determine the fracture type. Univariate and multivariate logistic regression analyses were used to identify the associated risk factors.

**Results:**

During the median follow-up period of 6 months, a total of 138 complications in 131 patients were determined, indicating the accumulated rate of 11.7%; there were 68 cases of secondary procedures, with the rate of 5.8%. The independent associated factors for postoperative overall complications were AO type C fracture (OR, 2.6; 95%CI, 1.2 to 4.0), open fracture (OR, 4.2; 95%CI, 1.9 to 6.5), and significant collapse of the lunate fossa (OR, 2.9; 95%CI, 13 to 4.3), and for secondary procedures were significant collapse of the lunate fossa (OR, 3.7; 95%CI, 1.7 to 6.4) and the low-volume of surgeons (OR, 95%CI, 1.2 to 3.6)

**Conclusions:**

Identification of these factors is of importance for the risk assessment of postoperative complications and the additional need of surgery. For patients with the above factors, especially those with combined risk factors, optimized operation scheme and high-volume surgeon should be considered to prevent or reduce the complications.

## Introduction

Distal radius fracture is the most common injury in department of emergency, trauma, or hand surgery. According to the epidemiological survey of traumatic fractures, distal radius fractures accounted for more than 4% of fractures in adults and up to 12% of fractures in the elderly [1], only behind hip fractures and spinal fractures. Although simple fall from standing height is the most common cause of such fracture, high-energy trauma such as motor accident, fall from a height, and industrial impaction injuries still account for a large proportion [2].

At present, open reduction and volar locking plate (VLP) fixation has become the standard surgical method for the treatment of such fractures and is superior to traditional conservative treatments [3, 4] and other methods [5–7]. However, multiple studies have reported a high incidence of postoperative complications following VLP fixation of distal radius fracture. Mckay et al. [8] found that the overall complications rate of the volar plate fixation of distal radius fractures ranged variedly, from 6 to 80%. The incidence of complications directly related to the plate was between 4% and 36% [9–11], and the secondary revision surgery rate was between 2% and 34% [12–14]. These complications not only affect the surgical results, but often require secondary operations, which prolong the hospital stay and increase the total costs of treatment [15, 16].

It is an important task for orthopedic surgeons to proactively evaluate the severity of fracture and patient surgical conditions to determine the risk of postoperative complications. Therefore, knowledge of the incidence of postoperative complications (each or overall) and identification of associated risk factors could be of specific importance in perioperative management. Moreover, it is the most cost-effective method to use these risk factors to screen high-risk patients and target the appropriate interventions to reduce the postoperative complications. However, to our best knowledge, only a few studies have evaluated the postoperative complications of VLP in the treatment of distal radius fracture [2, 16, 17], and some clinical factors such as the collapse of the lunate fossa have not been studied well.

The purpose of this study was to explore the incidence rate of postoperative complications following VLP fixation of distal radius fractures and to identify the independent factors of the overall complications and the secondary procedures.

## Patients and methods

### Inclusion and exclusion criteria

This was a retrospective study, approved by the ethics committee of The Third Hospital of Hebei Medical University. Between January 2013 and September 2018, patients with distal radius fractures who underwent volar plate fixation in our hospital were included in this study. Electronic medical record (EMR) and picture archiving and communication system (PACS) were inquired to extract perioperative data. Inclusion criteria were (1) age of 16 years and older, (2) definite diagnosis of distal radius fracture by radiograph or computed tomography (CT) scanning, and (3) treatment by VLP fixation. Exclusion criteria were (1) old fractures (> 3 weeks since fracture occurrence) or pathological (tumor metastasis) fracture, (2) treatments other than VLP, (4) patients who referred to another hospital for treatment of complications, and (5) patients who were lost to follow-up due to contact information change or due to personal affairs.

### Complications

These complications were obtained from the patient EMR, and the outpatient follow-up records and patients’ re-hospitalized EMR for treatment of complications. These complications included carpal tunnel syndrome (CTS), malreduction, loss of reduction, surgical site infection, plate loosening, intra-articular screw penetration, too long screws causing symptoms, nerve injury or irritation, blood vessel injury, extensor tendon or flexor tendon irritation/damage, complex regional pain syndrome type I (CRPS), re-fracture, fracture malunion, nonunion, traumatic osteoarthritis, and others. We defined the case where there was intra-articular screw penetration along with a restricted range of motion during the follow-up period as the complication of intra-articular screw penetration. Tendon irritation was diagnosed based on patients’ complaint of sore feeling when tendon was moving, with marked tenderness and swelling above the plate of the volar surface or the dorsal screws, or ultrasound showing the measurable liquid dark area and the increased synovial fluid at the metal hardware and tendon contact area. Nerve irritation was diagnosed based on patients’ complaint of hand or finger numbness or tingling, or the positive result of tapping or pressing test of nerve that there was neuroradiation or increased numbness. Secondary operations included carpal tunnel release, plate/screw removal ahead of schedule, debridement due to wound issue, re-fixation, skin grafting, tendon repair, transfer or reconstruction, osteotomy, and others.

### Perioperative variables

Two investigators inquired the EMRs and the PACS for data collection. These data include demographic and injury-related data, such as gender, age, height and weight (body mass index, BMI), place of residence (urban or rural area), injury mechanism, side, type of injury (closed or open), concomitant injury, AO classification, collapse of the lunate fossa, or existence of independent bone segment (< 3 mm × 3 mm); lifestyle and comorbidities, such as smoking, alcohol drinking, diabetes, hypertension, rheumatic immune diseases, and others; and surgery-related data, such as preoperative waiting time interval between fracture occurrence and the surgical fixation, American Society of Anesthesiologists (ASA) classification, anesthesia type, timing of surgery (emergency or elective surgery), the volume of the surgeon to treat, intraoperative blood loss, operation time, and postoperative drainage.

### Variable definition and grouping

BMI (kg/m^2^) was divided into five groups according to the criteria suited to Chinese population: underweight (< 18.5), normal (18.5–23.9), overweight (24.0–27.9), obesity (28.0–31.9), and morbid obesity (> 32.0) [18]. Preoperative waiting was divided into three groups: 0–3 days, 4–7 days, and > 7 days. The injury mechanism was further classified as low-energy and high-energy injury; the former referred to a fracture caused by a fall from standing height, and the latter referred to a fall from a height, motor accidents, mechanical injuries, or others. During this study period, surgeons who performed VLP of wrist fractures were ranked, based on the number of surgeries. A surgeon was classified as high volume if he performed the VLP fixation ≥ 35 cases (80th percentile among the surgeons), distinguished from those who performed surgery on 34 or fewer wrists (low-volume surgeon). Ninety-five minutes (80th surgical time among the full dataset), which was needed for VLP fixation distal radius fractures, was defined as the cutoff point, and exceeding this was defined as prolonged surgical duration.

### Statistical analysis

Mann Whitney *U* test or Student *t* test was used to evaluate the differences of continuous variables, when appropriate. Chi-square or Fisher’s exact test was used to evaluate the differences of categorical variables, when appropriate. Variables which were tested as approximately significant (*p* < 0.10) in the univariate analyses were entered into the multivariate logistic regression model to determine their independent effect, using stepwise backward elimination approach to exclude confounding covariates. Covariates with statistical significance (*p* < 0.05) were retained in the final model, and odds ratio (OR) and 95% confidence interval (95% CI) were calculated to indicate the correlation magnitude. The goodness-of-fit of the final model was evaluated by Hosmer–Lemeshow test and *p* > 0.05 represented an acceptable result. SPSS19.0 (IBM Corporation, Armonk, New York, USA) was used to perform all the analyses.

## Results

From January 2013 to September 2018, a total of 1962 patients with distal radius fractures were treated surgically in our hospital, and 1378 of whom were treated with VLP fixation (1406 distal radius fractures). Based on the inclusion and exclusion criteria, 1152 patient with 1175 fractures were in this study for data analysis. Of them, 964 (82.0%) were diagnosed by both X-rays and CT scanning preoperatively, and 106 (9.0%) underwent CT scanning at immediately postoperative period or during the follow-up periods. There were 432 male patients and 720 female patients, with an average age of 52.0 years (standard deviation, SD; 15.5 years; range, 16–83 years). There were 71 open fractures and 1104 closed fractures. The mean waiting time for surgical treatment was 3.3 days (SD, 2.2 days; range, 0–27 days).

During the study period, 131 patients had postoperative complications (138 cases), with the overall complication rate of 11.7% (138/1175). The most common complication was carpal tunnel syndrome (31, 2.6%), followed by mal-reduction or loss of reduction (23, 2.0%), wound infection (18, 1.5%), tendon irritation/rupture (15, 1.3%), CRPS (11, 0.9%), and others (Table 1). The earliest diagnosis of complication was at the first day after surgery, which was a case of superficial infection with redness, swelling, and pain in the wound site. After oral administration of a single dose of cephalosporin, the symptoms were relieved and disappeared.

**Table 1:** Complications of VLP for treatment of distal radial fractures

Complication	Number (incidence rate, %)
Carpal tunnel syndrome (CTS)	31 (2.6)
Mal-reduction or loss of reduction	23 (2.0)
Wound infection	18 (1.5)
Tendon irritation/rupture	15 (1.3)
Complex regional pain syndrome type I (CRPS)	11 (0.9)
Nerve irritation/adhesion	8 (0.7)
Plate loosening	6 (0.5)
Too long screw causing symptoms	6 (0.5)
Intra-articular screw penetration	4 (0.3)
Malunion	3 (0.3)
Non-union	2 (0.2)
Traumatic osteoarthritis	2 (0.2)
Others	9 (0.8)
Total	138/1175 (11.7)

There were 68 cases of secondary procedures, and the incidence rate was 5.8% (68/1175). The most common procedure type was carpal tunnel release (19, 1.6%), followed by re-fixation of plate/screw (14, 1.2%), plate/screw removal ahead of schedule (8, 0.7%), wound debridement (6, 0.5%) and, others (Table 2).

**Table 2:** Secondary procedure after VLP fixation for distal radial fractures

Secondary procedure	Number (incidence rate, %)
Carpal tunnel release	19 (1.6)
Re-fixation of plate/screw	14 (1.2)
Plate/screw removal ahead of schedule	8 (0.7)
Wound debridement	6 (0.5)
Tendon transfer	4 (0.3)
Ulnar shortening osteotomy	2 (0.2)
Skin grafting	3 (0.3)
Others	12 (1.0)
Total	68/1175 (5.8)

**Fig. 1:**
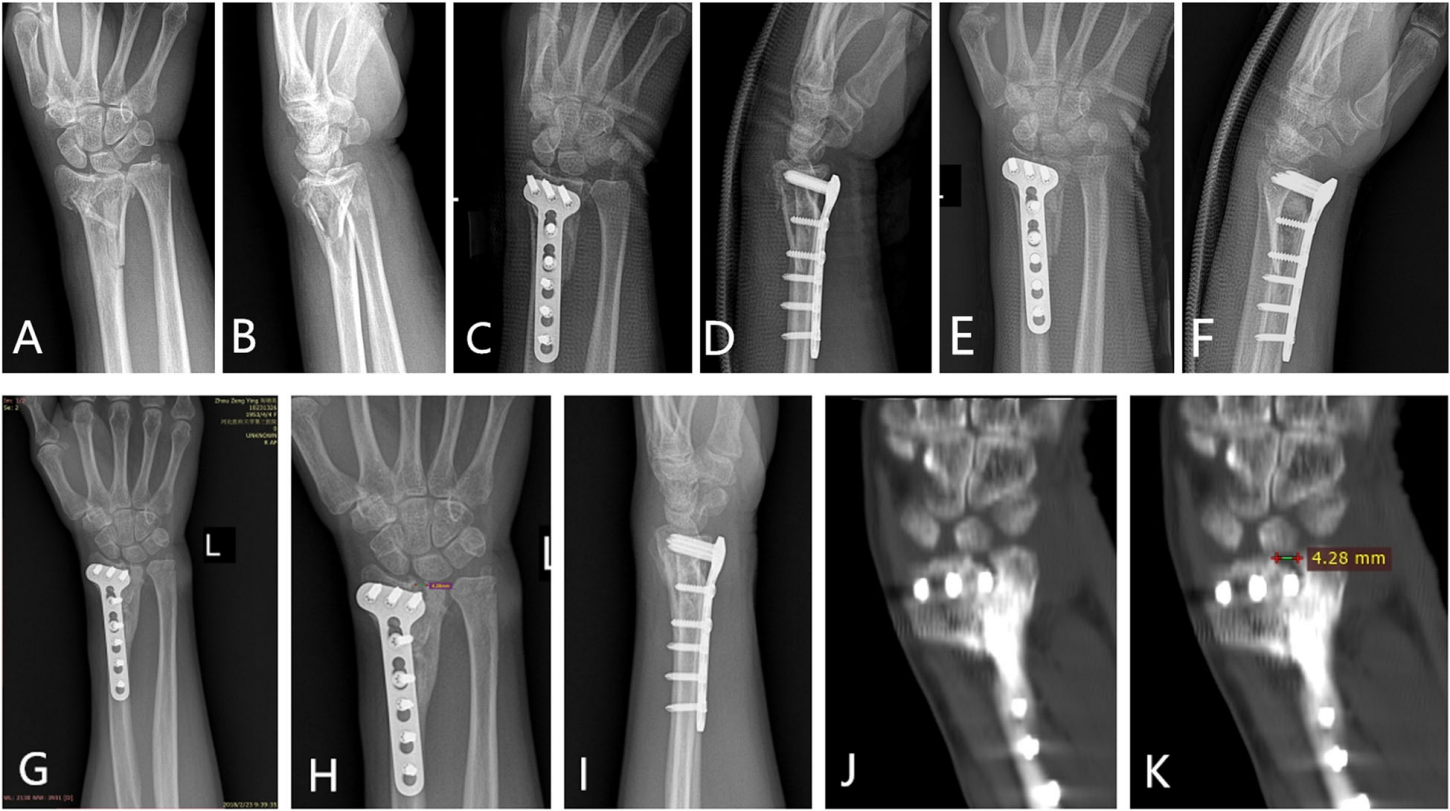
A 57-year woman had her left wrist fractured in an electric bicycle collision injury. The preoperative X-ray (**a** and **b**) showed AO type C2 fracture of distal radius. The immediate postoperative X-rays (**c** and **d**) showed the acceptable reduction and fixation. Postoperative 1-month X-rays (**e** and **f**) showed no evidence of loss of reduction. The postoperative 6-month X-rays and CT scanning showed obvious articular surface gap formation (4.28 mm)

**Fig. 2:**
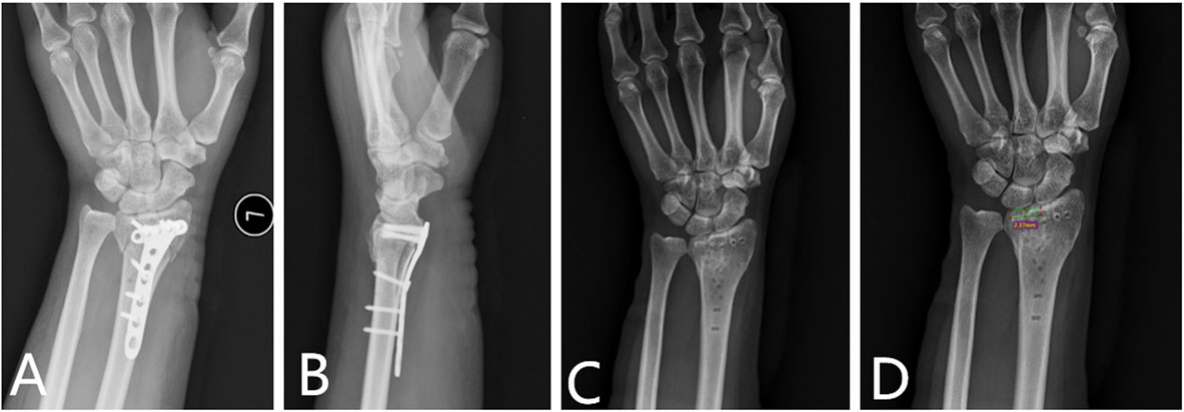
The immediate postoperative X-rays (**a** and **b**) showed good reduction and VLP fixation in a 44-year woman who had left wrist fracture (AO type B1). The X-rays (**c** and **d**) after removal of VLP at postoperative 14 months showed collapsed of the lunate fossa (2.37 mm)

Figures 1 and 2 showed 2 typical cases of loss of reduction involving the lunate fossa leading to articular step-off and the late displacement leading to articular surface gap formation.

### Risk factors for overall complications

Univariate analysis showed the significant differences between the two groups in term of age, living area, BMI, fracture type, AO classification of fracture, significant collapse of the lunate fossa, timing of surgery, and volume of surgeon (*p* < 0.05) (Table 2). The multivariate logistics regression analyses showed that AO type C fracture, open fracture, and significant collapse of the lunate fossa were independently associated with postoperative overall complications, as shown in Table 3. The Hosmer-Lemeshow test showed that the fitting degree of the final model was good (*X*^2^ = 6.714, *p* = 0.277, Nagelkerke *R*^2^ = 0.336)

**Table 3:** Univariate analysis of overall complications or secondary procedure following VLP fixation of distal radial fractures (138/1175)

Variable	Group	Rate of overall complication (%)	*p*	Rate of secondary-procedure (%)	*p*
Age (years)			0.040		0.438
	16–45	68/466 (14.6)		32/466 (6.9)	
	46–64	51/492 (10.4)		25/492 (5.1)	
	≥ 65	19/217 (8.8)		11/217 (5.1)	
Gender			0.082		0.386
	Male	60/443 (13.5)		29/443 (6.5)	
	Female	78/732 (10.7)		39/732 (5.3)	
Living area			0.048		0.256
	Living in urban area	54/376 (14.4)		26/376 (6.9)	
	Living in rural area	84/799 (10.5)		42/799 (5.3)	
BMI (kg/m^2^)			<0.001		0.239
	18.5–23.9	63/534 (11.8)		29/534 (5.4)	
	< 18.5	11/39 (28.2)		4/39 (10.3)	
	24.0–27.9	24/369 (7)		16/369 (4.3)	
	28.0–31.9	32/177 (13.5)		15/177 (8.5)	
	≥ 32.0	8/56 (34.8)		4/56 (7.1)	
Smoking			0.300		0.226
	Yes	43/321 (13.4)		23/321 (7.2)	
	No	95/854 (11.2)		45/848 (5.3)	
Alcohol			0.361		0.351
	Yes	39/372 (9.4)		25/372 (6.7)	
	No	99/803 (7.9)		43/803 (5.4)	
Diabetes			0.155		0.092
	Yes	9/67 (13.4)		7/67 (10.4)	
	No	93/1108 (8.4)		61/1108 (5.5)	
Hypertension			0.183		0.508
	Yes	24/256 (9.4)		17/256 (6.6)	
	No	114/919 (12.4)		51/919 (5.5)	
Rheumatoid diseases			0.494		0.037
	Yes	7/47 (14.9)		6/47 (12.8)	
	No	131/1128 (11.6)		62/1128 (5.5)	
Injury mechanism			0.147		0.048
	Low energy	86/796 (10.8)		39/796 (4.9)	
	High energy	52/379 (13.7)		29/379 (7.7)	
AO classification			< 0.001		0.020
	Type A	21/264 (8)		11/264 (6.1)	
	Type B	38/428 (8.9)		18/428 (4.9)	
	Type C	79/483 (16.4)		39/483 (6.4)	
Fracture type			0.011		< 0.001
	Open	14/64 (21.9)		12/64 (18.8)	
	Closed	124/1101 (11.3)		56/1101 (5.1)	
Significant collapse of the lunate fossa			0.001		< 0.001
	Yes	13/48 (27.1)		10/48 (20.8)	
	No	125/1127 (11.1)		58/1127 (5.1)	
Concomitant fractures			0.147		0.074
	Yes	13/77 (16.9)		8/77 (10.4)	
	No	125/1098 (11.4)		60/1098 (5.5)	
Volume of treatment of these fractures (years)			0.023		0.040
	High (≥ 35)	79/774 (10.2)		37/774 (4.8)	
	Low (< 35)	59/401 (14.7)		31/401 (7.7)	
Preoperative stay (days)			0.280		0.135
	0–3	59/517 (11.4)		28/517 (5.4)	
	4–7	47/442 (10.6)		21/442 (4.8)	
	> 7	32/216 (14.8)		19/216 (8.8)	
Timing of surgery			0.016		0.092
	Emergency	43/271 (17.4)		10/271 (3.7)	
	Elective	95/904 (12.2)		58/904 (6.4)	
ASA classification			0.206		0.266
	I–II	97/884 (11)		55/884 (6.2)	
	≥ III	41/291 (14.1)		13/291 (4.5)	
Surgical duration (mins)			0.057		0.092
	< 95	102/940 (10.9)		49/940 (5.2)	
	≥ 95	36/235 (15.3)		19/235 (8.1)	
Intraoperative blood loss (ml)			0.549		0.013
	< 200	100/876 (11.4)		42/876 (4.8)	
	≥ 200	38/299 (12.7)		26/299 (8.7)	
Postoperative drainage			0.260		0.858
	Yes	54/403 (13.4)		24/403 (6)	
	No	84/772 (10.9)		44/772 (5.7)	

*Abbreviation: OR* odds ratio, *VLP* volar locking plate, *BMI* body mass index, *AO* Arbeitsgemeinschaftfür Osteosynthesefragen, *ASA* American Society of Anesthesiologists

### Risk factors for a secondary procedure

There were significant differences between the two groups in term of rheumatoid disease, injury mechanism, fracture type (open), AO classification of fracture, significant collapse of the lunate fossa, intraoperative blood loss, and volume of surgeon (*p* < 0.05) (Table 2). The multivariate logistics regression analyses showed that a significant collapse of the lunate fossa and low volume of surgeons were independently associated with a secondary procedure, as shown in Table 4. The Hosmer-Lemeshow test demonstrated the adequate fitness of the final model (*X*^2^ = 7.244, *p* = 0.219, Nagelkerke; *R*^2^ = 0.391)

**Table 4:** Multivariate logistic regression analysis of risk factors for overall complications or secondary procedure following VLP fixation of distal radius fractures

	OR and 95% CI	*p* value
Overall complications		
AO type C fracture	2.6 (1.2 to 4.0)	0.02
Open fracture (vs closed)	4.2 (1.9 to 6.5)	< 0.001
Significant collapse of the lunate fossa	2.9 (1.3 to 4.3)	0.001
Secondary procedure		
Significant collapse of the lunate fossa	3.7 (1.7 to 6.4)	< 0.001
Low-volume of surgeon	2.5 (1.2 to 3.6)	0.018

*Abbreviation: OR* odds ratio, *VLP* volar locking plate, *AO* Arbeitsgemeinschaftfür Osteosynthesefragen

## Discussion

Postoperative complications following VLP fixation of distal radius fractures are common and intractable, which seriously affect functional recovery and increase the treatment costs. This study summarized the data from patients’ EMRs during the past over 5 years to investigate the incidence and risk factors associated with postoperative overall complications and the secondary procedure. The results showed the incidence of overall complications is 11.7%, and the independent associated risk factors were AO type C fracture, open fracture, and the significant collapse of the lunate fossa. The incidence rate of a secondary procedure was 5.8%, and the independent associated factors were a significant collapse of the lunate fossa and low volume of surgeons.

In previous studies, the incidence of postoperative overall complications of VLP fixation of distal radius fractures varied widely, ranging from 4 to 36%, depending on the study design, the participants, sample size, and the follow-up period [9, 11, 19]. In a recent meta-analysis, Bentohami et al. [9] included 33 original studies and found that the overall complication rate was 16.5%, slightly higher than ours. The authors concluded the most common complication was impaired nerve or tendon, which was confirmed as the third most common complication in this study. The possible explanation maybe that all the original studies included in the meta-analysis were prospective studies, which were more likely to capture the functional problems such as tendon and nerve damage during the follow-up period.

Both the severity of fracture and the damage of soft tissue will increase the risk of postoperative complications. In this study, it was shown that open injury and AO type C fracture were independently associated with these complications. Sirnio et al. [16] studied 881 cases of distal radius fractures treated by VLP and found that open fracture was associated with a higher risk of postoperative complications (9% vs 7%), although the result did not reach statistical significance. In that study, it was speculated that the smaller size sample was related to the non-significant result because there were only 22 open fractures included. Glueck et al. [20] included 42 cases of open distal radius fractures and suggested the degree of wound contamination was significantly related to the postoperative infections, but not the classification level according to Gustilo and Anderson or Swanson system. Tsang et al. [21] found the rate of complications following VLP for treatment of AO type C distal radius fractures was 22.2%, which was slightly higher than ours (16.4%); the authors found the non-significantly different complication rate between volar and dorsal surgical approach (25% vs 19.0). We recommend that type C fractures especially with contamination should be treated with multiple and thorough debridements as part of the initial treatment plan to reduce or prevent the complications.

In our study, we found that the significant collapse of the lunate fossa (5 mm or more) were independent factors associated with the increased risk of overall complications and the need of a secondary procedure. According to anatomical and imaging studies, the lunate fossa accounted for 52 to 53% of the articular surface of the distal radius, which is the central axis of the loading on the wrist joint surface. Therefore, lunate fovea collapse or poor reduction will seriously affect the movement of the wrist joint [22, 23]. On the other hand, the lunate fossa collapse fracture is often caused by high-energy impact injury, known as die-punch fracture, which is difficult to reduce and also a challenge to maintain the stability after its reduction. We previously studied 93 type B distal radius fractures fixed by VLP, with a significant lunate fossa collapse in 21 cases and non-significant in 72 cases, and found the significantly different rate of “articular step-off” (19% vs 4%). Similarly, Beck et al. [24] demonstrated the significant collapse of the lunate fossa (5 mm or more) and the volar cortex length available for fixation less than 15 mm were independent predictors for postoperative reduction loss.

Loss of reduction involving the lunate fossa is a frequently encountered complication, and one direct result is the formation of articular step-off. In this study, 23 cases of mal-reduction or loss of reduction were noted; of them, eight involved the lunate fossa. As we described above, fractures involving the lunate fossa are difficult to reduce because the volar surface of the distal radius slopes in a volar and ulnar direction [22]. A standard volar plate fixation is insufficient to provide equal fixation and stabilization to both the scaphoid and the lunate fossa. Harness et al. [25] reported that seven patients with initial anatomic reduction all developed loss of reduction of the volar lunate fragment during the postoperative period. Berglund et al. [26] presented a typical case that inadequate fixation of the lunate fossa caused postoperative loss of reduction, even if the adequate initial reduction was obtained. Alternative to standard volar plate, fragment-specific fixation might be a better choice due to its specific stabilization of individual intra-articular fracture fragment [27, 28]. The auxiliary fixation by Kirschner wires was also a preferred choice, but the risk of damage to the superficial radial nerve should be considered [29]. In addition, the bone quality and the position of distal locking screws in the subchondral region should also be taken into consideration, because they were correlated with progressive late displacement [30].

Low volume of surgeons for the treatment of complex distal radius fracture was also demonstrated to increase the risk of a secondary procedure, but not for the overall complications. However, the role of volume of surgeons for operations remains controversial. In the study by Ward et al. [17], the authors reported that the complications were significantly reduced in surgeons who had more than 30 cases of experience in the treatment of distal radius fractures. Sirnio et al. [16] found that high seniority of surgeons (20 cases of distal radius fracture or more) was associated with lower incidence of complications than low seniority (less than 20 cases) (4% vs 10%). In contrast, Soong et al. [2] found high seniority (20 cases of distal radius fracture or more) was associated with higher late complication rate (e.g., traumatic arthritis) than lower seniority (less than 20 cases) in their study of 594 distal radius fractures. In our institution, the treatment allocation of patients in our hospital is mainly based on the severity of injury, and the randomness is not so adequate. Even so, we still observed the significant difference rate of a secondary procedure between high-volume and low-volume surgeons. Therefore, we suggest that complex injuries especially those type C fractures and lunate bone depression collapse should be managed by a more experienced surgeon, so as to reduce the occurrence of complications.

One of the limitations of this study was the retrospective design, which compromised the accuracy of the data collection. Secondly, we cannot quantify some variables, for the number of cigarettes each day and the duration, which might have a dose effect on postoperative complications. Thirdly, we could not obtain the information on the functional recovery of the wrist joint in patients who had a complication requiring additional surgical procedure. Fourthly, it should be noted that the relationship between these complications and VLP might not be causal, but at least correlative. Therefore, they should be treated cautiously. The future prospective studies are needed to validate our results and to continue the investigation of the subsequent effects of complications or revision procedure.

In conclusion, we found the incidence rate of overall complication was 11.7% and identified several risk factors associated with the increased risk of overall complications, including open fracture, type C fracture and a significant collapse of the lunate fossa. The incidence rate of a secondary procedure was 5.8%, and the independent associated factors were significant collapse of the lunate fossa and low volume of surgeons. Almost all of these factors were not modifiable, but they do aid in predicting the potential complications and counseling patients and their relatives regarding the risk of the procedure. The future technique or studies should focus on how to more effectively manage the significantly collapsed fracture segments of the lunate fossa.

## Data Availability

All the data will be available upon motivated request to the corresponding author of the present paper.

## Abbreviations

AO: Arbeitsgemeinschaftfür Osteosynthesefragen
ASA: American Society of Anesthesiologists
BMI: Body mass index
CRPS: Complex regional pain syndrome
CT: Computed tomography
CTS: Carpal tunnel syndrome
EMR: Electronic medical records
OR: Odds ratio
PACS: Picture archiving and communication system
SD: Standard deviation
VLP: Volar locking plate
